# Factors Influencing Antibiotic Prescribing Behavior and Understanding of Antimicrobial Resistance Among Veterinarians in Assam, India

**DOI:** 10.3389/fvets.2022.864813

**Published:** 2022-04-26

**Authors:** Mahmoud Eltholth, Gurrappanaidu Govindaraj, Banani Das, M. B. Shanabhoga, H. M. Swamy, Abin Thomas, Jennifer Cole, Bibek R. Shome, Mark A. Holmes, Dominic Moran

**Affiliations:** ^1^Global Academy of Agriculture and Food Security, The Royal (Dick) School of Veterinary Studies and the Roslin Institute, The University of Edinburgh, Edinburgh, United Kingdom; ^2^Hygiene and Preventive Medicine Department, Faculty of Veterinary Medicine, Kafrelsheikh University, Kafr El-Shaikh, Egypt; ^3^ICAR-National Institute of Veterinary Epidemiology and Disease Informatics (NIVEDI) Ramagondanahalli, Bangalore, India; ^4^Department of Health Studies, Royal Holloway, University of London, Egham, United Kingdom; ^5^Department of Veterinary Medicine, University of Cambridge, Cambridge, United Kingdom

**Keywords:** antibiotics, antimicrobial resistance, livestock, veterinarians, prescribing, behavior

## Abstract

This study investigates factors influencing veterinarians' antibiotic prescribing behaviors and their understanding of antimicrobial resistance (AMR). The study used a telephone survey of 50 veterinarians conducted in five districts in Assam state, India. The survey sought information on the most prevalent animal diseases, veterinarians' awareness of potential preventive measures, including factors determining antimicrobial prescribing; the types of antimicrobials used for different health conditions in different species, and possible options to reduce antimicrobial use (AMU). The majority (86%) of respondents worked for the government, 98% reported having no written policy for the use of veterinary health products, and 58% have no on-site diagnostic facilities. Ceftriaxone, Enrofloxacin, and Oxytetracycline were the antibiotics (ABX) most frequently prescribed, by 76, 68, and 54% of veterinarians, respectively. These ABX were prescribed mainly for respiratory health problems and mastitis in cattle, and gastrointestinal infections in buffaloes, sheep, goat, and pigs. Severity of clinical symptoms, economic status of the livestock owner, and withdrawal period for ABX were ranked as very important factors for giving ABX. Less than two thirds (64%) were aware of the government ban for Colistin and only 2% were aware of a national plan for AMR. This study highlighted that ABX prescription is mostly based on tentative diagnosis given the lack of diagnostic facilities in most veterinary clinics. There is a need to enhance veterinary healthcare and to improve communication between policy makers and field veterinarians and, importantly, a need to disseminate clear prescribing guidelines on prudent AMU.

## Introduction

Antimicrobial use (AMU) in livestock production has beneficial impacts on animal health and welfare, and on household livelihoods, food security, and food safety (1, 2). However, there is increasing concern about the role of livestock production in the emergence of antimicrobial resistance (AMR) and consequent human exposure to resistant pathogens and/or antibiotic residues through the food chain, direct contact with livestock, or indirectly, via a contaminated environment (3–6). The loss of antibiotic efficacy due to AMR is a global public health challenge and a complex problem driven by many interconnected biological and socio-economic factors. Consequently, isolated interventions and non-coordinated actions may have limited impact on its management. Instead, effective mitigation of AMR risks requires dialogue and coordinated engagement between stakeholders from public and private sectors along the animal and antibiotics (ABX) value chains, implicating veterinarians, public health scientists, microbiologists, environmental scientists (ecologists), agricultural/forestry scientists, and epidemiologists (2, 7–9). Broader regulatory oversight is required, including of importation, distribution, sale, and use (7, 10). This regulatory complexity is part of a quintessential One Health (OH) approach implying the need to coordinate action across human, animal and environmental settings.

In the animal setting, veterinarians are key stakeholders in terms of diagnosis and treatment decisions for livestock. Yet, the international evidence on veterinary antimicrobial stewardship (AMS) is conflicting. Even in developed countries like Australia, small companion animal veterinarians reported prescribing broad-spectrum ABX of higher importance to human health more frequently than was the case for livestock veterinarians (11). The same study observed that the cost of bacterial culture to identify the causative agent, antimicrobial susceptibility testing (AST), and the lack of access to rapid and affordable diagnostics were barriers to appropriate ABX prescribing. A systematic review for non-clinical factors influencing veterinarians' prescribing behavior of ABX (12) indicated that fear of possible complications or losing customers if ABX were not prescribed (identified in 19 out of 34 studies), self-confidence (19/34), business factors (19/34), and complacency (16/34) are influencing veterinarians' decisions. Other influencing factors were; lack of awareness of animal owners regarding AMS (16/34) and demand for ABX (12/34), lack of appropriate regulations (10/34), cost and time for culture and AST (10/34), and inadequate farm hygiene (8/34) (12) with AMU as a “quick fix” to cover for poor hygiene and biosecurity practices (13).

There are conspicuous evidence gaps in low and middle-income countries (LMICs), where pharmaceutical regulation continues to be weak and diagnosis hampered by poor access to laboratory tests, poor training, negligible awareness, and lack of biosecurity measures (12, 14). Other factors influencing AMU are a higher burden of animal diseases such as mastitis in dairy cattle (15, 16). Identifying and understanding these barriers and drivers is important for designing strategies for rationalizing AMU in livestock production (12). A survey of Nigerian veterinarians revealed that only 21% were able to define the term “antimicrobial stewardship,” and 60% were unaware of the National Action Plan guidelines for AMR. More than half perceived prophylactic AMU as appropriate under poor farm biosecurity, and only 20% frequently conducted AST before deciding to administer ABX for treatment (17). A similar situation pertains in known global AMR hotspots, such as China and India, where livestock production is projected to grow in line with income growth and consumption preferences (18), and where there is an urgent need to evaluate the feasibility and acceptability of veterinary AMU control measures.

Accordingly, this study aimed to assess factors influencing antimicrobial prescribing behavior of a sample of veterinarians in Assam, India, where access to medicines is poorly regulated (19–21). The output of this study would inform the design of potential AMR control interventions, and to bridge the gap between prescribing guidelines and clinical usage. The research builds on evidence indicating that Indian veterinarians liberally prescribed ABX for animal cases based on the initial diagnosis, and that there is no strong relationship with farmers to ensure active involvement of stakeholders in more rational AMU (7, 9, 19, 22).

## Methods

### Study Area and Data Collection

A purposive sampling method was used to interview veterinarians from Kamrup, Nagaon, Dhubri, Mangaldloi, and Dhemaji districts of Assam and from the state capital Guwahati. Contact details of veterinarians were obtained from the Animal Husbandry and Veterinary Department (AHVD). Due to COVID-19 restrictions, it was not possible to conduct face-to-face interviews, so all interviews were conducted by telephone; 100% of veterinarians in the list had telephone number. A structured questionnaire was used to collect socio-demographic data on veterinarians, and further information was collected on livestock health management, the most prevalent livestock health problems, factors influencing AMU and understanding of AMR, and potential interventions (the survey questionnaire is available as Supplementary Material 1). Questions used a 3-point Likert scale to assess levels of (dis)agreement with statements (1 = Disagree, 2 = Neutral, 3 = Agree and 99 = Don't know), and a 5-point Likert scale to rank items in order of importance (1 = Not important; 2 = Slightly important; 3 = Fairly important; 4 = Important; 5 = Very important). The survey also used open-ended questions probing ABX use and veterinarians' awareness and understanding of opportunities for reducing AMU in livestock production.

The questionnaire was piloted with five veterinarians working at the College of Veterinary Science, Assam Agricultural University. It was developed in English and conducted by dual lingual English/Assamese speaking members of the research team. The questionnaire was the only data collection tool used for this study.

### Data Management and Analysis

The enumerators filled in hard copies of the questionnaire during the telephone call to avoid any recall bias. Data were stored electronically in a Microsoft Excel database and anonymised for further analysis. Statistical analyses were conducted using Excel and SPSS Statistics v24.0 (IBM SPSS Statistics for Windows Version 24.0, New York: IBM Corp). The Likert scale questions were analyzed using mean and mode values, and standard deviations. Mean values were used to summarize questions to which most respondents agreed or disagreed (23). Verbatim responses for some open-ended questions were summarized without rephrasing.

## Results

### Demographic Information

The characteristics of veterinarians participated in this survey (*n* = 50) are summarized in Table 1. The average age was 27.96 (*SD* = 4.63) years and the number of males (56%) was higher than females (44%). A high proportion (64%) had 3–10 years of experience and the majority (86%) were working for the AHVD, Government of Assam. Only 4% reported selling veterinary health products in addition to providing other veterinary services, probably in private clinics. Almost all (98%) veterinarians reported that they didn't have a written policy for the use of veterinary health products, and 58% have no diagnostic facilities in their clinics. The distance to the nearest diagnostic facility ranged from 0.2 to 40 km with an average of 2.91 km and a mode of 1 km.

**Table 1:** Characteristics of veterinarians who participated in the survey.

**Characteristics**	**Value**
**Age, years**	
Min Max Mean, SD	23 46 27.96 (4.63)
**Gender**, n*(%)	
M F	28 (56) 22 (44)
**Education**, ***n*** **(%)**	
BVSc MVSc PhD	23 (46) 22 (44) 5 (10)
**Years of experience**, ***n*** **(%)**	
<3 years 3–10 years More than 10 years	16 (32) 32 (64) 2 (4)
**Work for**, ***n*** **(%)**	
Own clinic Government Private clinic	2 (4) 43 (86) 5 (10)

^*^n, number.

### Prevalent Diseases and Factors Influencing AMU

In cattle, parasitic diseases (PD), mastitis, Foot and Mouth Disease (FMD), and Lumpy Skin Disease (LSD), were reported as the most prevalent diseases by 74, 50, 44, and 40% of veterinarians, respectively. In buffaloes, FMD and ketosis were reported by 16 and 12% of veterinarians, respectively. In goats, PD, Peste des petits ruminants (PPR), Listeriosis and Orf were reported by 42, 40, 24, and 24% of veterinarians, respectively. In pigs, FMD and PD were reported by 16 and 10% of veterinarians, respectively (Supplementary Table 1). The top five health problems in poultry were Newcastle disease (ND), *E. coli*/Colibacillosis, Salmonellosis, Infectious Bursal Disease (IBD), and Fowl Pox reported by 64, 20, 12, 10, and 10% of veterinarians, respectively (Supplementary Table 2). Respondents suggested a range of preventive measures for prevalent diseases and health conditions in different animal species, Supplementary Table 3. Regular vaccination was highly recommended for FMD in cattle, PPR in goat, Classical Swine Fever (CSF) in pigs, and ND in poultry by 48, 40, 48, and 64% of respondents, respectively. Deworming was recommended for PD in cattle, buffaloes, goat and pigs by 68, 16, 40, and 10% of veterinarians, respectively. Proper management was highly recommended for the prevention of mastitis in cattle by 28% of respondents.

The average daily number of cases investigated by a veterinarian was highest in poultry 12.32 (Median = 4, *SD* = 19.17) followed by cattle 6.34 (Median = 4, *SD* = 6.23), Table 2. In addition to treatment of diseased animals, veterinarians participated in other activities such as awareness campaigns/programmes for farmers (58%), insurance (36%), castration (18%), and artificial insemination 12%.

**Table 2:** Animal species and number of cases per day investigated by veterinarians.

**Animal species**	**Number of vets (%)**	**Cases per day**		
		**Mean**	**Median**	**SD**
Cattle	50 (100)	6.34	4	6.23
Buffaloes	17 (34)	4.47	2	6.96
Sheep	7 (14)	3.43	2	3.21
Goat	48 (96)	4.73	3	4.89
Poultry	47 (94)	12.32	4	19.17
Pigs	37 (74)	4.51	2	6.68

**Figure 1:**
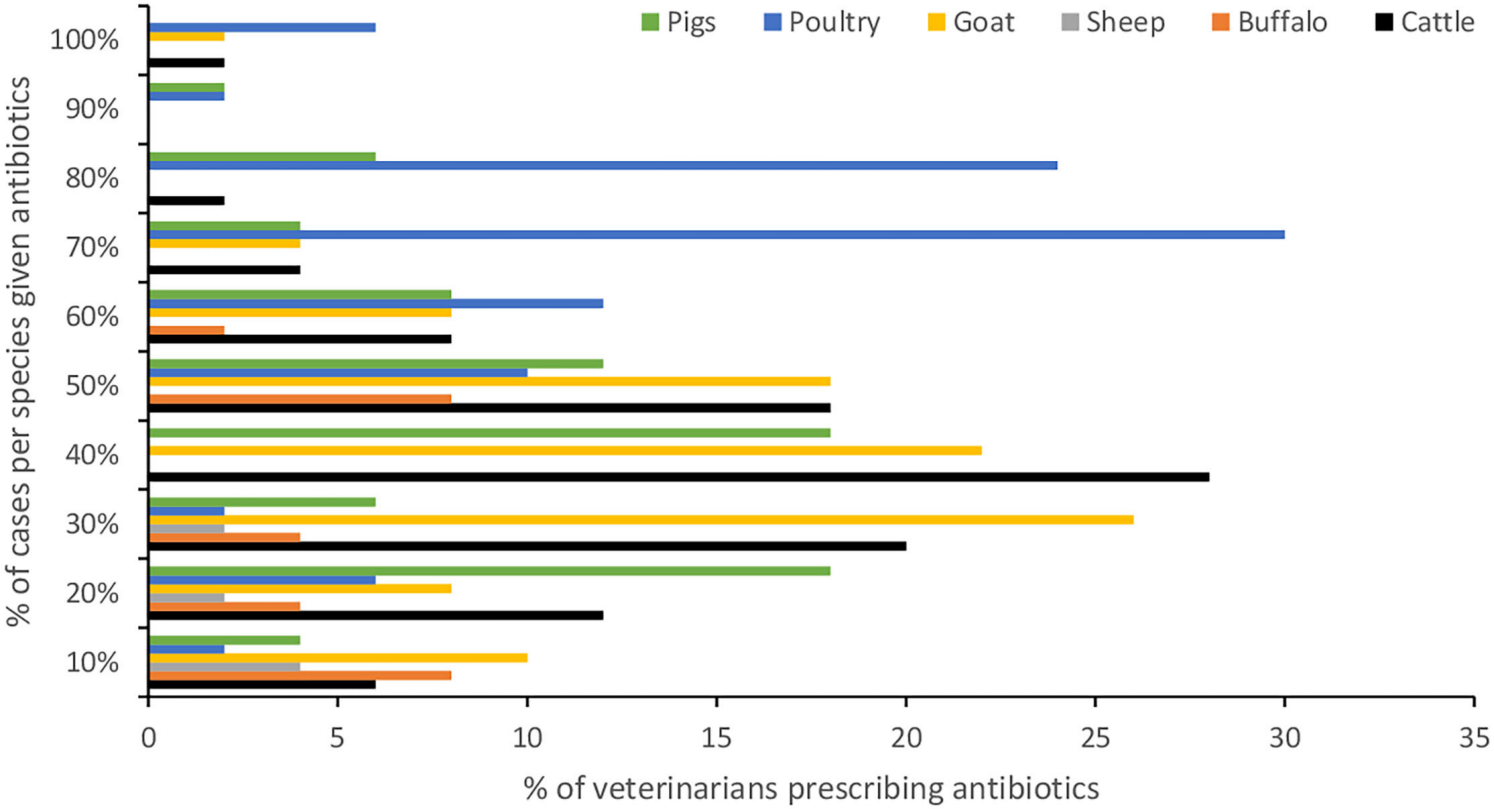
Proportion of cases of different animal species for which antibiotics were prescribed by veterinarians.

**Figure 2:**
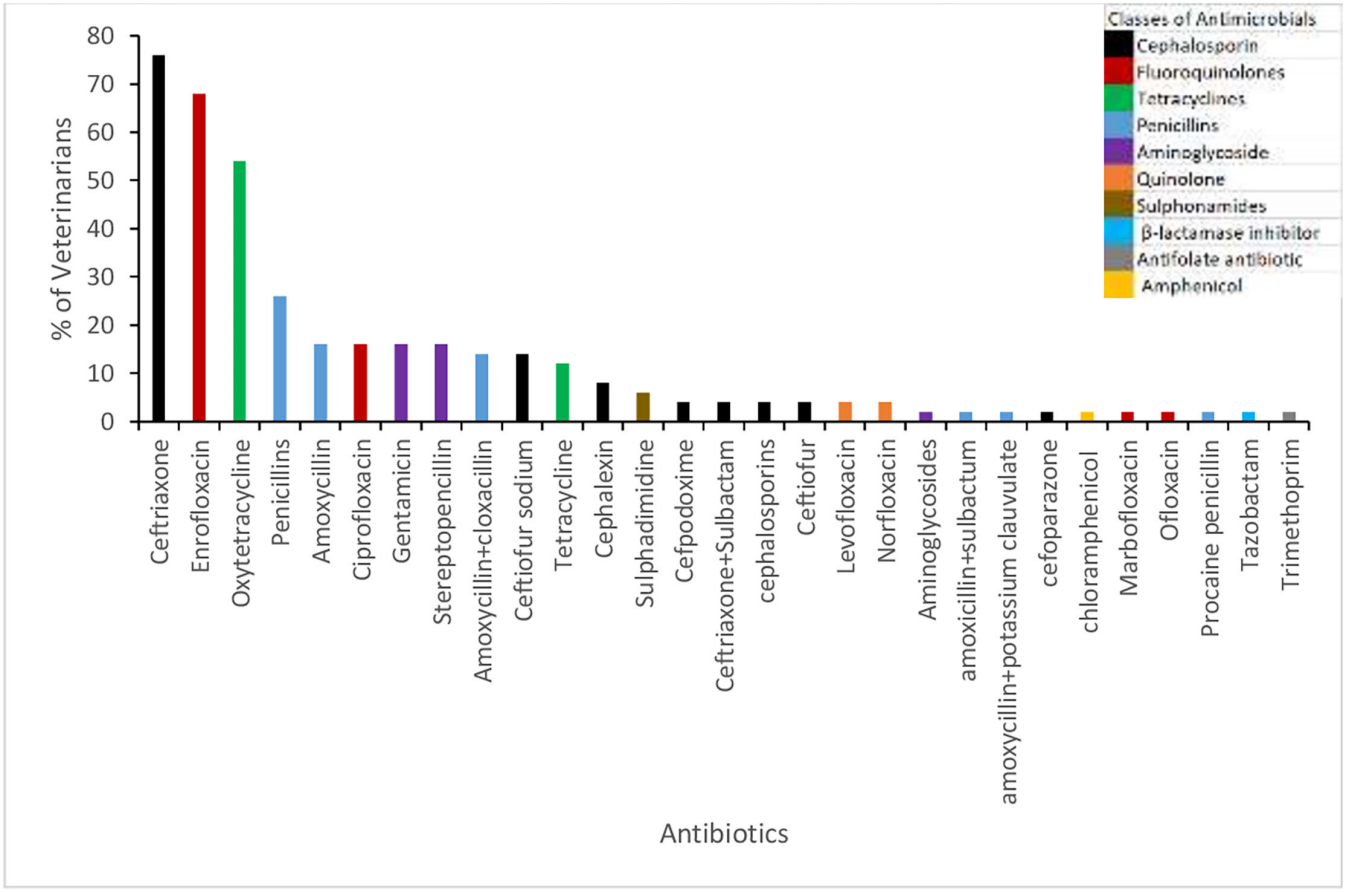
Types of antibiotics prescribed by veterinarians.

Almost all respondents (96%) stated that they prescribe ABX for animals with health problems. The percentage of veterinarians and the proportion of cases per animal species for which ABX were prescribed are shown in Figure 1 (veterinarians were asked “out of 10 cases for how many do you prescribe ABX?”). In cattle, over the 3 months prior to the interviews a high proportion of veterinarians (88%) reported using ABX for respiratory health problems, followed by mastitis (82%). The average number of cases of gastrointestinal tract infections (42.58) and mastitis (14.59) were higher in cattle compared with other animal species. The highest average number of cases of lameness was in buffaloes (34.50), Table 3. Ceftriaxone, Enrofloxacin, and Oxytetracycline were the top ABX reportedly prescribed by 76, 68, and 54% of veterinarians, respectively (Figure 2).

**Table 3:** Average number of cases for different health problems given antibiotics over the last 3 months.

**Health problem**	**Cattle**		**Buffaloes**		**Sheep/Goat**		**Pigs**	
	**% of veterinarians**	**Average No. of cases**	**% of veterinarians**	**Average No. of cases**	**% of veterinarians**	**Average No. of cases**	**% of veterinarians**	**Average No. of cases**
Gastrointestinal	76.00	**42.58**	**30.00**	19.93	**84.00**	**29.60**	**60.00**	**7.63**
Respiratory	**88.00**	21.93	26.00	13.85	68.00	21.65	50.00	6.56
Lameness	38.00	12.53	12.00	**34.50**	40.00	7.80	20.00	3.50
Reproductive	76.00	12.00	14.00	10.86	68.00	10.35	30.00	4.87
Mastitis	82.00	**14.59**	14.00	10.43	56.00	8.82	16.00	6.13

*Bold values are the highest values*.

**Figure 3:**
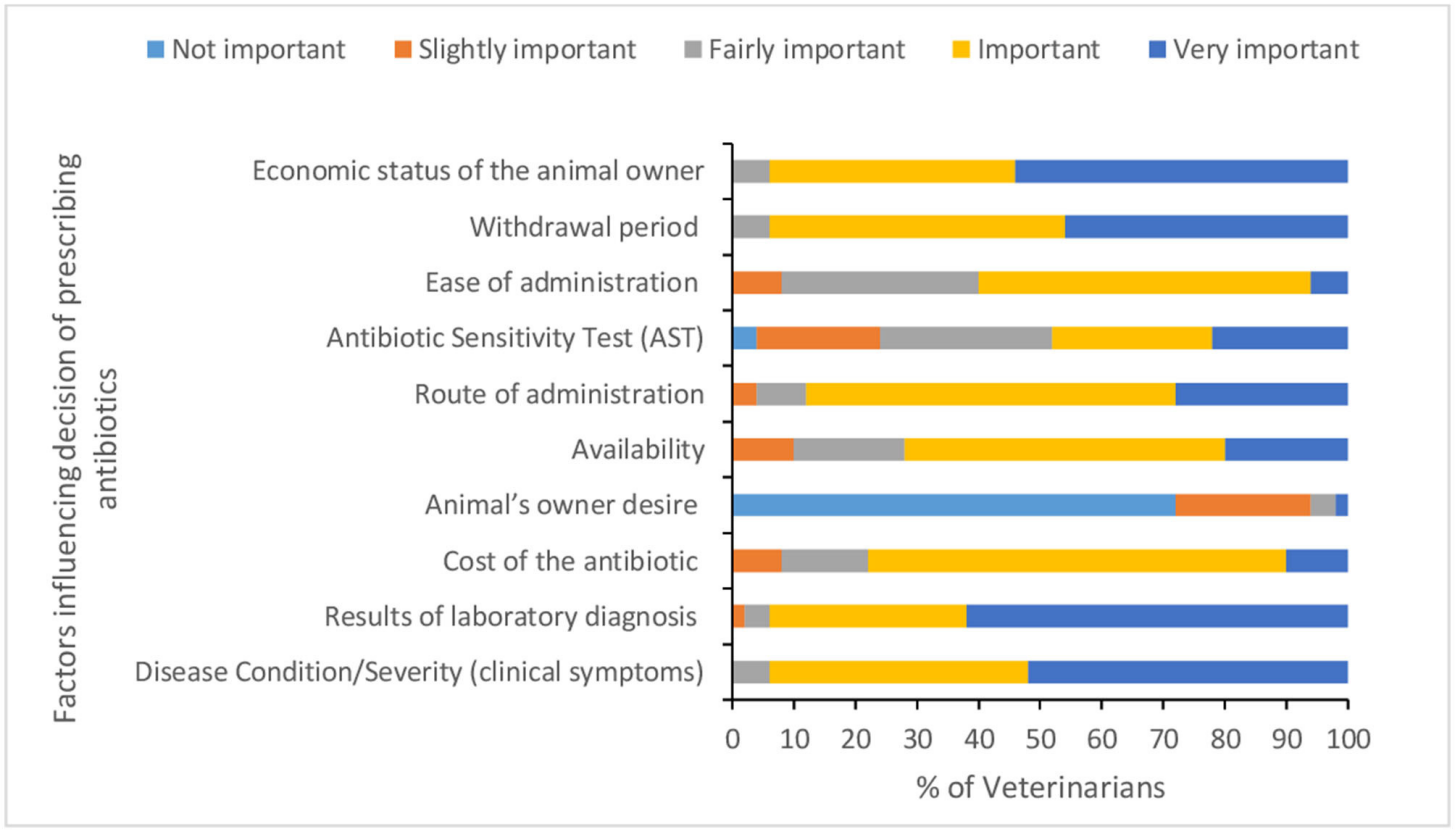
Factors influencing the selection of antibiotics by the veterinarians.

**Figure 4:**
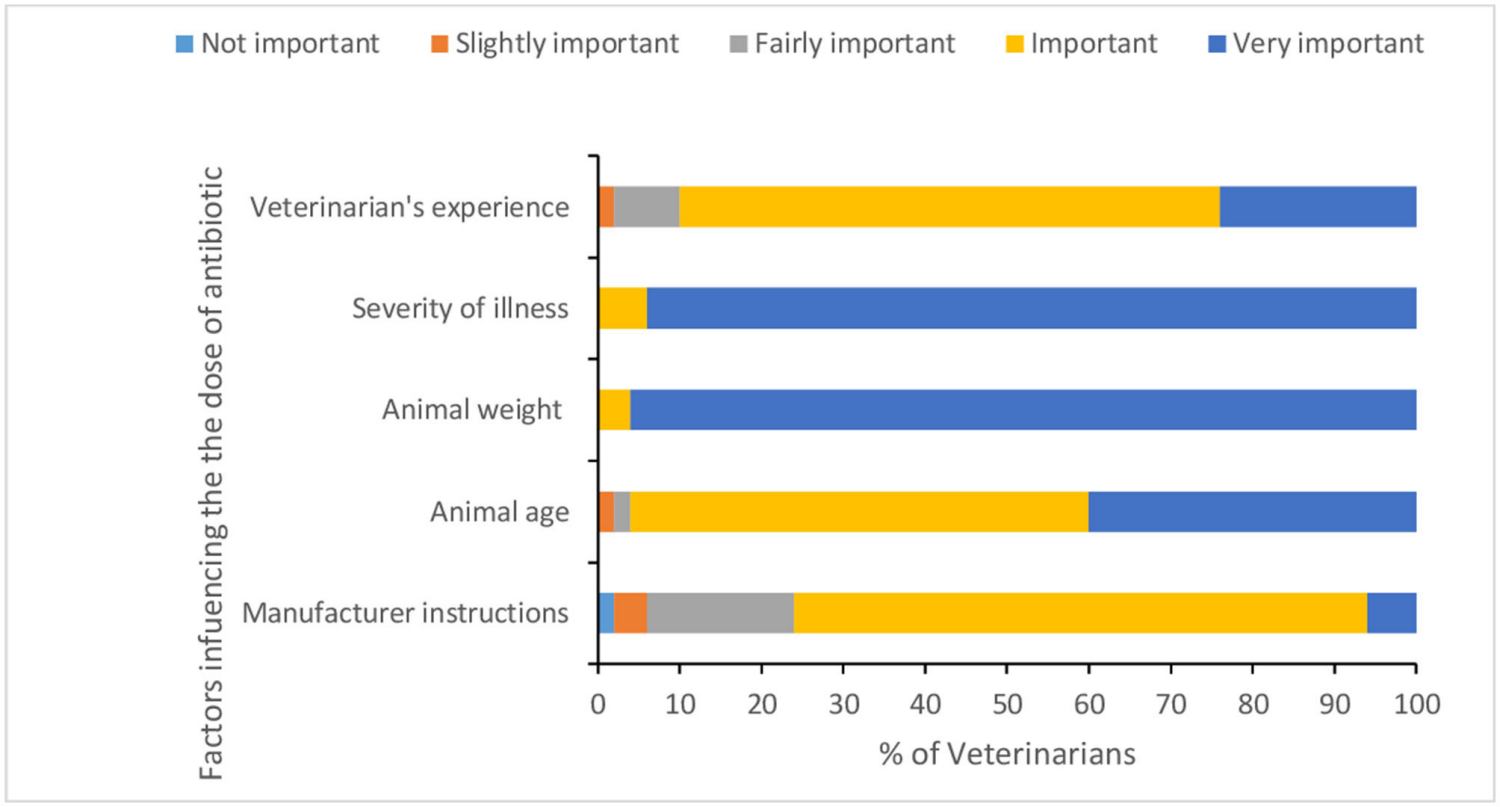
Factors influencing veterinarians' decisions for antibiotic dose.

Fifty six percent of veterinarians reported performing surgery for animals, of which 10.71% sometimes give ABX as prophylactic for 1–2 days before surgery. However, the majority of veterinarians (97.7%) reported always giving ABX post-surgery for 3–7 days with an average of 4.68 (*SD* = 0.98) days. Specific ABX reported as used before surgery were Enrox^®^, Cefotaxin^®^, Xyrofur^®^, Ciprofloxacin, Amoxycillin, and Intacef-Tazo^®^. Less than one third of veterinarians reported conducting AST and taking samples for bacterial culture before AMU. Disease condition/severity of clinical symptoms, results of laboratory diagnosis, economic status of the animal owner, and withdrawal period were reported as very important factors influencing decision for prescribing ABX by 52, 62, 54, and 46% of veterinarians, respectively (Figure 3 and Supplementary Table 4). The main factors for calculating the dose of ABX (Figure 4 and Supplementary Table 4) were animal weight, severity of illness, and animal age. Only one veterinarian indicated that all of his clients complete the recommended course of ABX, while 26% of veterinarians indicated 70% of their clients complete the recommended course of ABX.

### Veterinarians' Understanding of AMR

**Figure 5:**
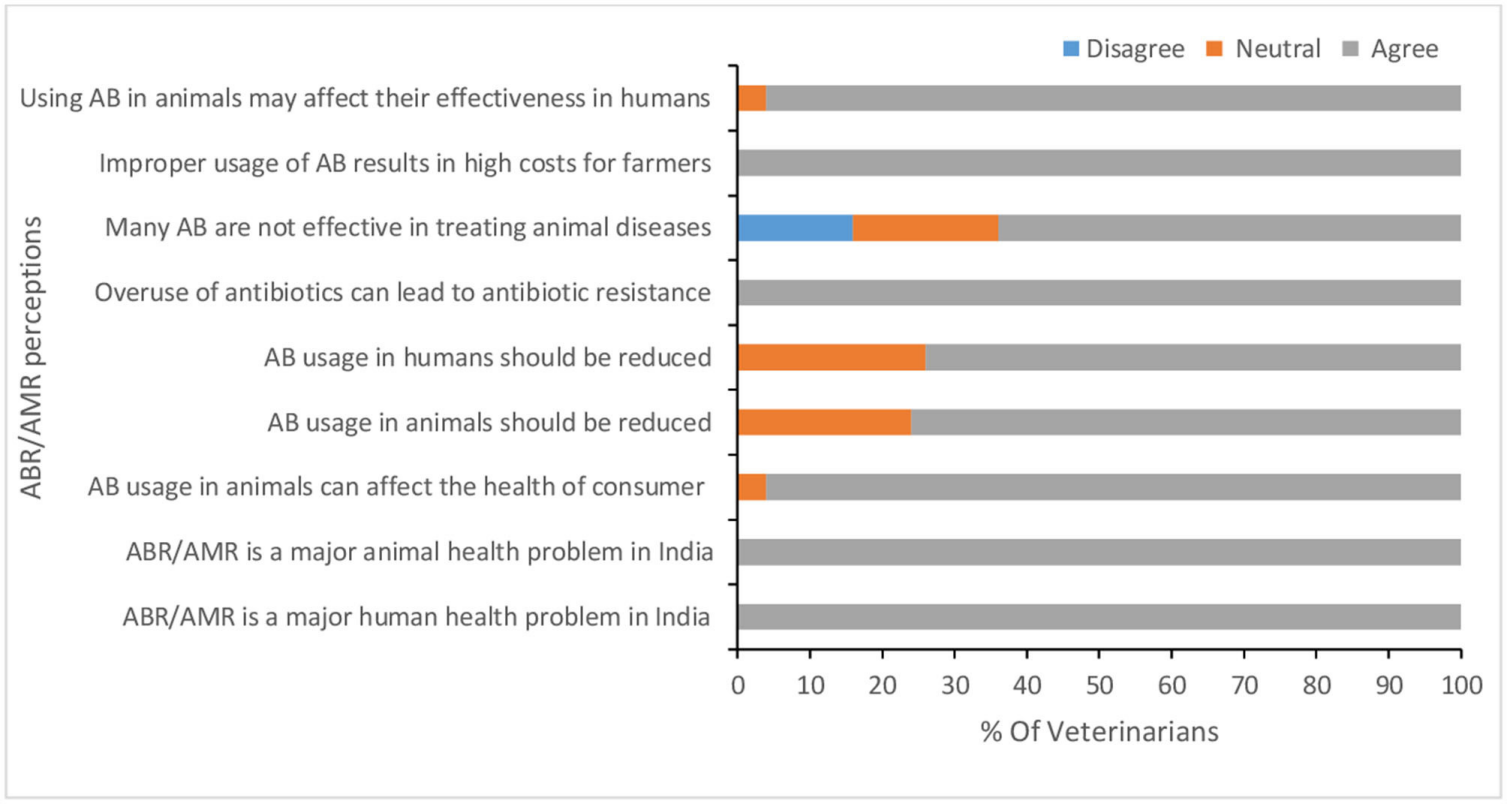
Veterinarians' perceptions of antibiotic resistance/antimicrobial resistance (AMR).

All veterinarians believed that ABX are effective and indicated that they have heard about AMR. The main source of their knowledge about AMR was from their undergraduate (BVSc) and postgraduate courses (MVSc/PhD). More than half of participants believe that it is possible to raise livestock without AMU, and 77% stated they prescribe alternatives to ABX; while 23% claimed that ABX alternatives are not easily available. Veterinarians' perceptions of AMU and AMR are summarized in Figure 5 and Supplementary Table 5. All veterinarians agreed with the statement that AMR is a major human and animal health problem caused by over use and/or improper use of ABX.

Less than two thirds (64%) of veterinarians were aware of the government ban for Colistin. The main reasons understood for the ban, amongst, amongst those who were aware of it, (Table 4) were “Due to development of resistance” (28%) and “To prevent AMR and Colistin is the ABX of last resort, so if humans become resistant to this there will be no ABX left to treat” (18.75%). Only 2% of respondents stated they are aware of a national and/or a state policy plan for AMU in livestock.

**Table 4:** Veterinarians' opinions for the reasons for the ban on Colistin use for animals in India.

**Reasons of banning Colistin**	**No**	**Percentage %**
**Due to development of resistance**	**9**	**28.13**
**To prevent AMR and Colistin is the last resort of antibiotic, so if humans become resistant to this there will be no antibiotic left to treat**	**6**	**18.75**
To reduce the Colistin residue which is a potential source of resistance	2	6.25
Residual effect of Colistin on humans	2	6.25
Once animal becomes resistant to Colistin, there is no cure to any disease	2	6.25
Hazardous/dangerous to human health	2	6.25
Hazardous to animal as well as human health	2	6.25
To stop use of this antibiotic	1	3.13
To stop antibiotic resistance in humans	1	3.13
To stop propagation and multiplication of antibiotic resistant bacteria and associated bacteria	1	3.13
To reduce antimicrobial resistance	1	3.13
If an animal is given antibiotic like Colistin and if humans consume that milk or meat then human becomes resistant to that antibiotic which is dangerous	1	3.13
As Colistin is the highest form of antibiotic, so if the animals become resistant to it, then it will be difficult to treat the animals further.	1	3.13
Due to adverse effect of Colistin on animals and humans	1	3.13

*Bold values are the highest values*.

### Veterinarians' Opinions for Prudent AMU and Controlling AMR

**Figure 6:**
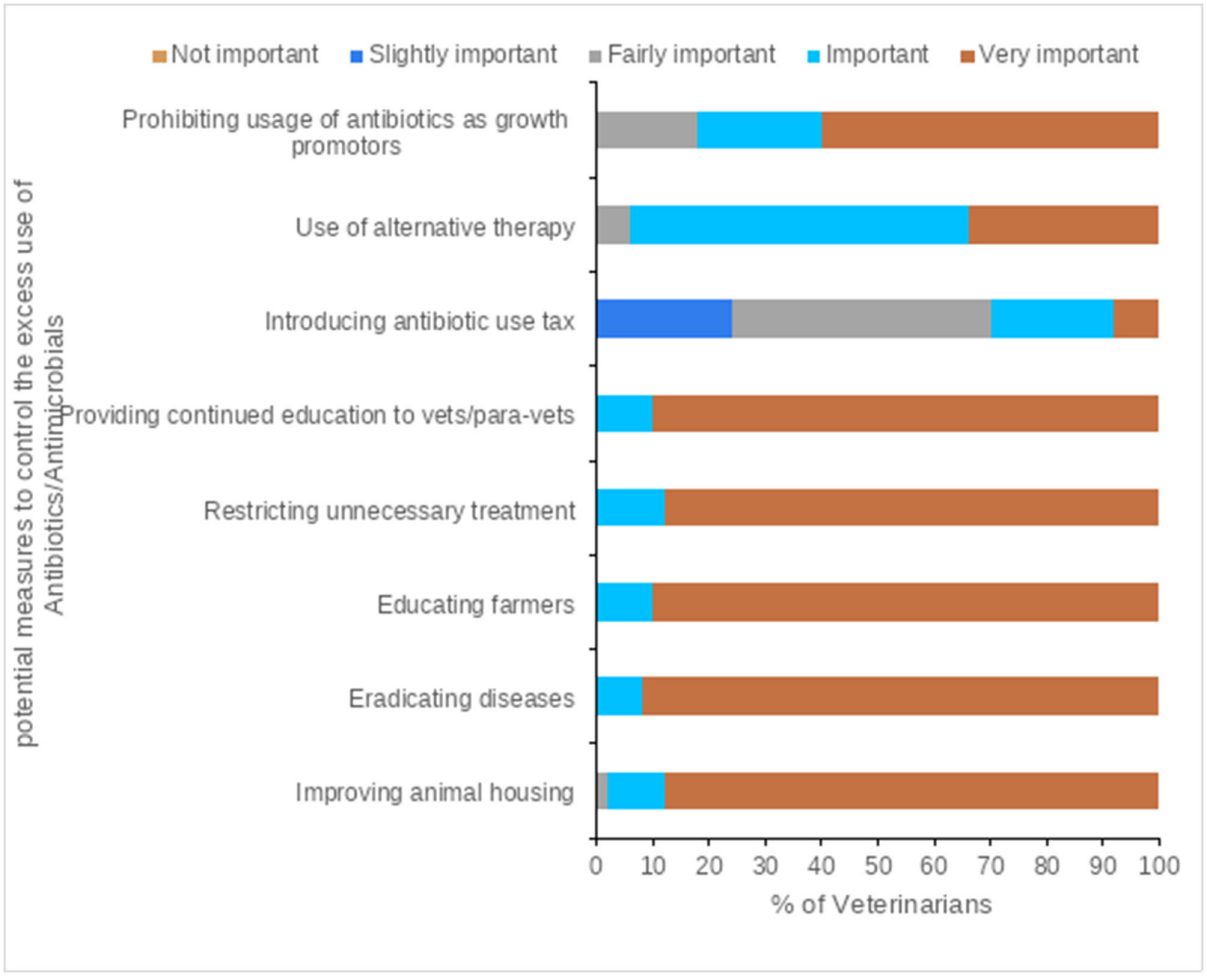
Veterinarians' attitudes to the potential measures to control the excess use of Antibiotics/Antimicrobials.

**Figure 7:**
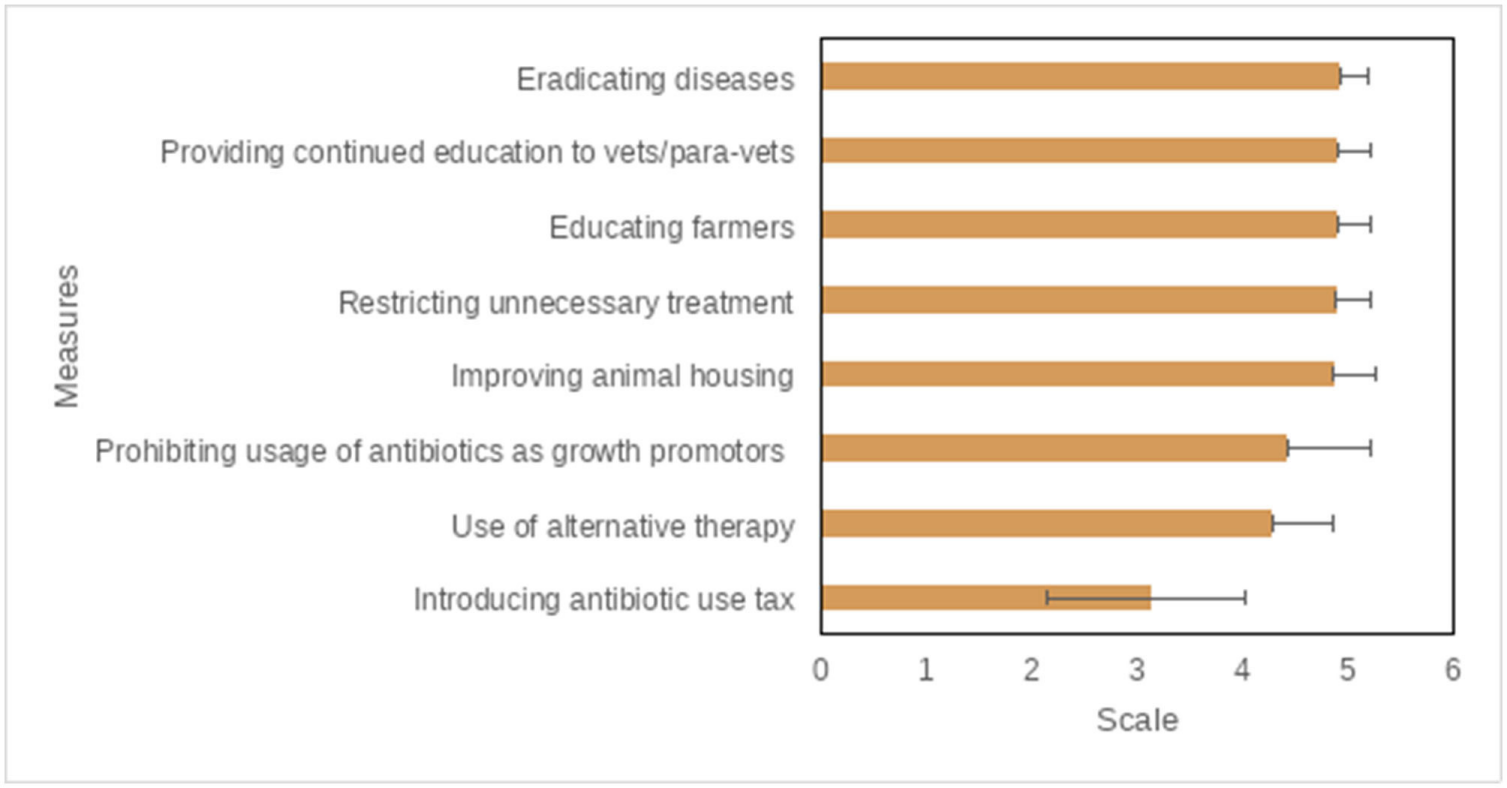
Potential measures to control the excess use of antibiotics/antimicrobials, questions were scored on a 5-point Likert scale (1 = Not important; 2 = Slightly important; 3 = Fairly important; 4 = Important; 5 = Very important), the mean score is displayed here from lowest to highest.

**Figure 8:**
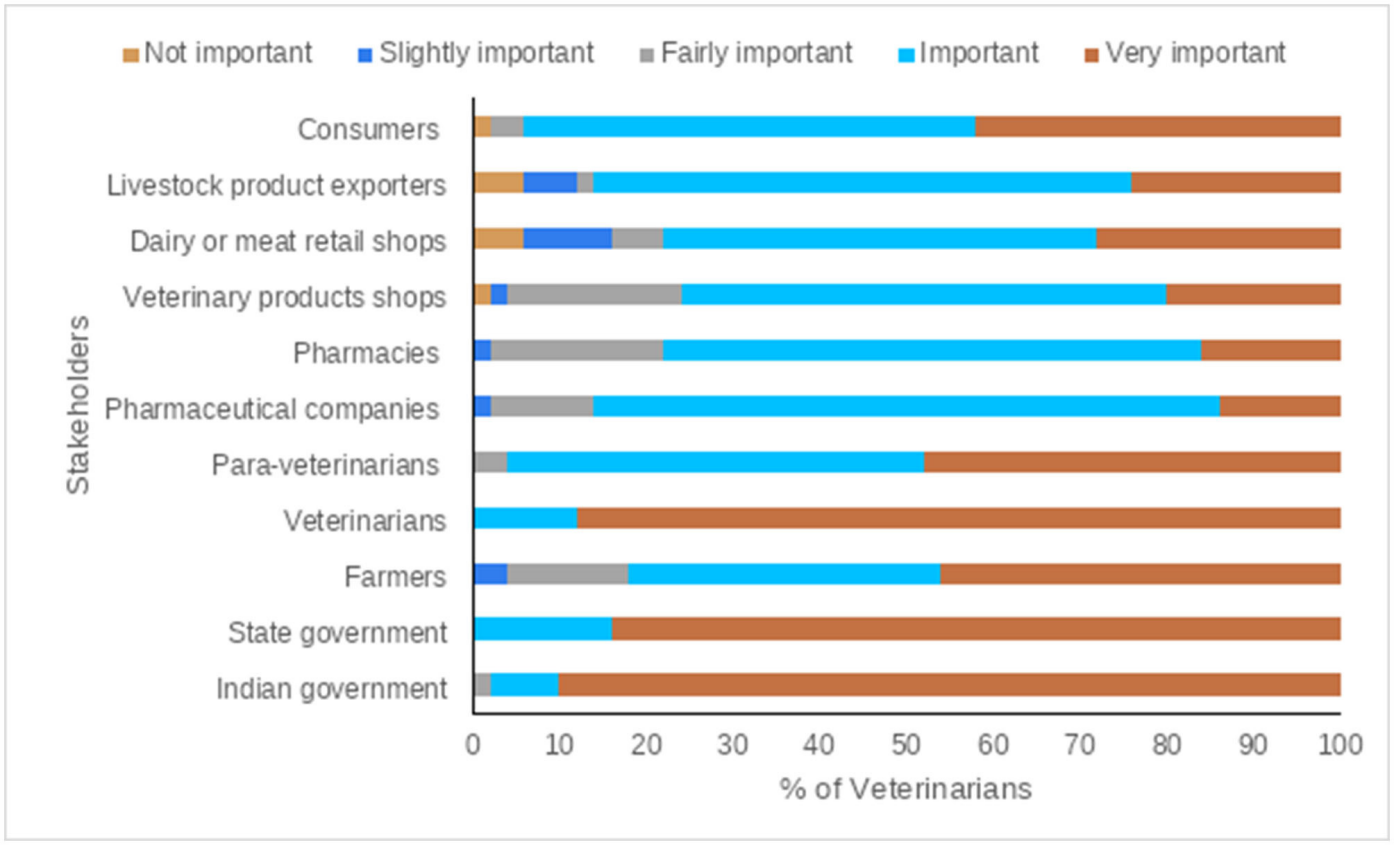
Veterinarians' opinions of the main stakeholders in the control the excess use of Antibiotics/Antimicrobials.

**Figure 9:**
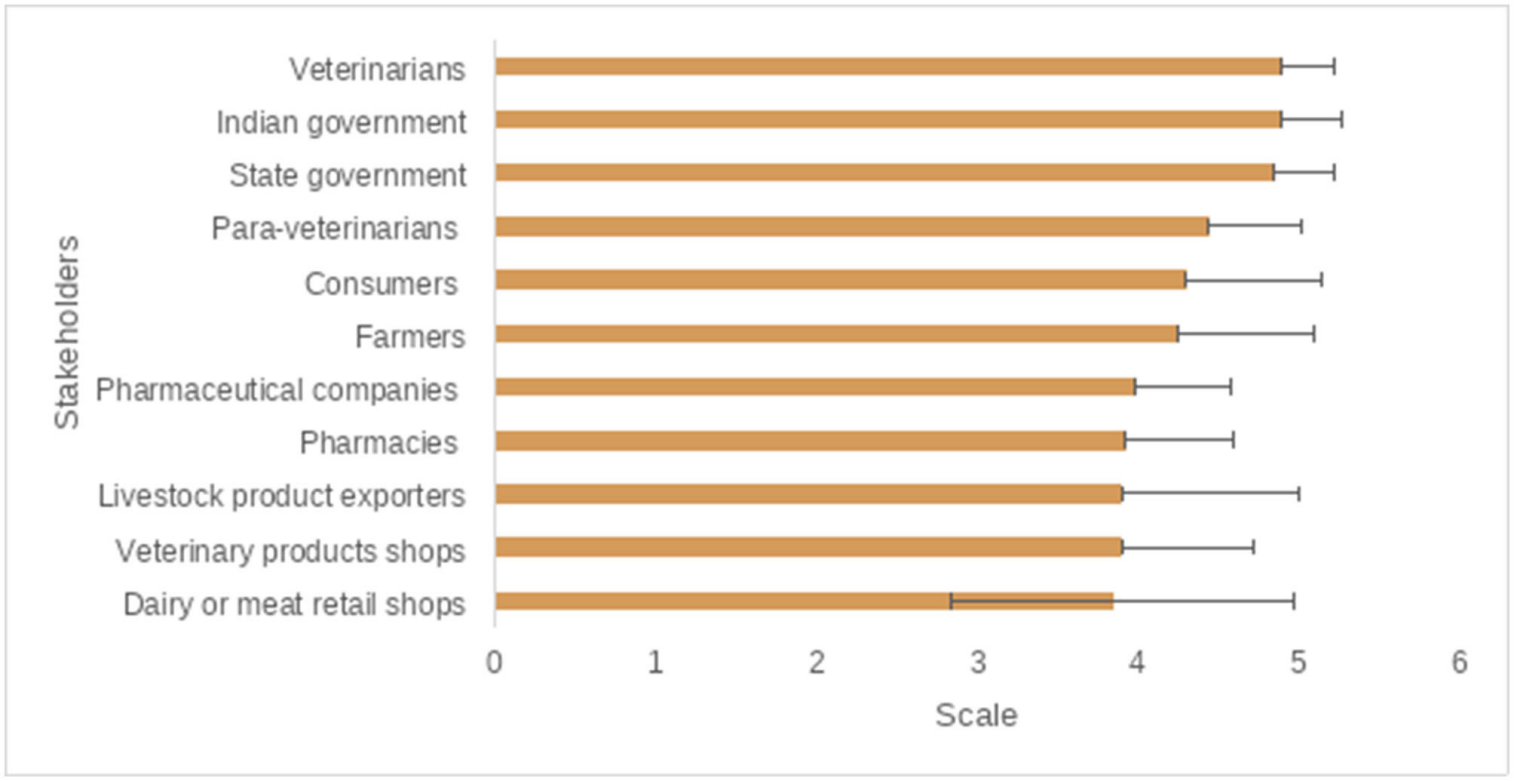
The main/key stakeholders to control the excess use of antibiotics/antimicrobials, questions were scored on a 5-poinet Likert scale (1 = Not important; 2 = Slightly important; 3 = Fairly important; 4 = Important; 5 = Very important), the mean score is displayed here from lowest to highest.

Continued education (for veterinarians, para veterinarians, and farmers), disease eradication, improving animal housing, and restricting unnecessary treatments were ranked as very important measures to control the excess AMU in livestock production by the majority of participants (Figures 6, 7, and Supplementary Table 6). Among others, central government, state governments, and veterinarians were ranked as very important key stakeholders to control the excess AMU, Figures 8, 9, and Supplementary Table 6.

Veterinarians suggested potential prophylactic measures for animal diseases to be able to raise animals without AMU, Table 5. The most frequently mentioned measures were vaccination, deworming, hygiene, and biosecurity, suggested by 88, 40, 32, and 28% of veterinarians, respectively.

**Table 5:** Potential prophylactic measures for animal diseases suggested by veterinarians.

**Potential prophylactic measures for animal diseases**	**No**.	**Percentage %**
**Vaccination**	**44**	**88**
**Deworming**	**20**	**40**
**Hygienic measurers**	**16**	**32**
**Biosecurity measures**	**14**	**28**
Good/proper management practices	9	18
Sanitation	7	14
Balanced diet	5	10
Proper feeding	4	8
Dietary management	2	4
Scientific rearing of animals	2	4
Quarantine of diseased animal	2	4

*Bold values are the highest values*.

The main suggestions to reduce ABR/AMR among the farming community (Table 6) were: farmers should consult veterinarians for any diseased animal (34%), complete the course of treatment (34%), and pay more attention to farm hygiene, sanitation, and management (20%). Other actions included establishment of diagnostic labs, using ABX alternatives, and proper animal nutrition.

**Table 6:** Veterinarian's suggestions for reducing antimicrobial resistance in farming community.

**Suggestions to reduce AMR among the farming community**	**No**.	**Percentage %**
**Farmers should consult the vet for any diseased animal**	**17**	**34**
**Antibiotic course should be completed**	**17**	**34**
**Farm hygiene, sanitation and management**	**10**	**20**
Herbal medicine first	5	10
Antibiotic awareness among farmers	5	10
Judicious use of ABX	5	10
Biosecurity	4	8
Proper dosing of ABX	4	8
Vaccination	3	6
Antimicrobial resistance awareness among farmers	3	6
Using narrow spectrum ABX first	2	4
Availability of cost effective ABX	2	4
Self-prescription medicines shouldn't be given by own	2	4
Quack practice should be stopped	2	4
Using ayurvedic medicines first then going for ABX is good	2	4

*Bold values are the highest values*.

### Training and Future Collaboration

Only 26% of veterinarians reported having attended at least one seminar/training workshop related to AMU in livestock and AMR since graduation from veterinary school. Training providers were mostly government organizations including AHVD of Assam, universities and research centers. Only one veterinarian was willing to keep a record of the veterinary health products prescribed for animals, for a 3 months prospective study, though 50% said they were possibly willing to participate.

## Discussion

The survey results indicate a lack of awareness of the national and/or state policy/plan, despite the existence of a national action plan for AMR (NAP-AMR 2017–2021),^1^ which among other aims seeks to improve “*awareness and understanding of AMR through effective communication, education and training*.” In Assam, the AHVD has a Professional Efficiency Development scheme to regulate veterinary practices according to the Indian Veterinary Council Act, 1984. The Council is also responsible for the development of training modules and continued training of field veterinarians. Another project, “Assistance to States for Control of Animal Diseases,” is also supporting the training needs of veterinarians, para-veterinarians as well as laboratory persons. However, the extent of AMU training and awareness in these is unclear and there is a need to improve collaboration between different organizations such as veterinary colleges and the AHVD (15, 19, 25), and for them to be aligned with the objectives of the NAP. Despite this, only 26% of the survey respondents reported having attended at least one training/awareness seminar since graduating however, suggesting that opportunities for continued professional development (CDP) are limited. Less than two thirds (64%) of participants were aware of the government ban for Colistin and the reasons for this decision in their opinions were varied. The verbatim responses (Table 4) showed a potential confusion or lack of understanding over whether it is human/animals who developed resistance to the antibiotics or the pathogens. Veterinarians might have used “animal/humans resistance” as a proxy or indicator for not responding to treatment with ABX. Core competencies among veterinarians have been found to be lacking or inefficient and it has been suggested that training and attending national and international seminars/workshops are appropriate ways to acquire competencies (26). There is a continuing education and training needs for veterinary students, working veterinarians, para-veterinarians and other stakeholders for which online and open and distance learning programmes in animal welfare need to be developed (27). In India and other LMICs with limited resources, private sector and non-governmental organizations (NGOs) could play an important role to support continuous education and training of veterinarians. “Vet Sustain” is an example of a UK-based social enterprise working to enable and inspire veterinary professionals to continually improve the health and wellbeing of animals, people and the environment. It is also supporting veterinary professionals to drive change toward a more sustainable future and to become leading forces for sustainability.^2^ Guidelines, provided by NGOs such as the British Veterinary Association, the British Small Animal Veterinary Association, and the British Cattle Veterinary Associations, have been considered a key step for appropriate AMU (28, 29). Such activities provide opportunities for keeping stakeholders up to date with the latest government advice and regulation, better ways to support training and CPD in India using a similar model could be investigated.

As found in previous studies (11, 30–32), low levels of laboratory diagnosis for animal diseases continues to be a factor in speculative AMU, and arises due to a lack of onsite diagnostic facilities. In this study, 58% of veterinary clinics have no diagnostic laboratories and 70% of veterinarians never use AST before AMU. The AHVD has a scheme for assisting states to set up infrastructure for new veterinary hospitals and dispensaries and to strengthen/equip the existing ones.^3^ However, coverage and access are still not universal for all states. In 2015–16, the contribution by the central government was increased from 60 to 90% for 11 states (8 North-East and 3 Himalayan states) which may improve the veterinary services in Assam. There are disease diagnostic laboratories only at the state and district level. A field veterinarian who wants to conduct basic tests has to send samples to these laboratories, which is time consuming. Hence, equipping the veterinary clinics and dispensaries with basic equipment including microscopes, on-farm diagnostic kits and other tools for screening some of the diseases will help field veterinarians to diagnose quickly and to prescribe appropriate ABX based on confirmed infection instead of depending on clinical signs alone. A survey of animal health professionals in 20 sub-Saharan African countries concluded that the development of on-farm diagnostics and management tools for animal diseases are essential to considerably improve AMU (32).

Respondents in this study reported that farmers often call and/or consult them only after trying other options. This can lead to the progression of health problems that are less likely to respond to medications and more likely to develop resistance. Non-responsive cases also drives a lack of confidence between farmers and veterinarians (15). This behavior might be due to the shortage of veterinarians in Assam; there are around 1,000 veterinarians but the required number is estimated at 2,529 (one veterinarian per 5,000 cattle head) according to the National Commission of Agriculture.^4^ Ethnographic work with farmers in Assam (conducted under this project but yet to be published) identified lack of access to veterinarians and veterinary services as a major barrier to proper management of animal health; farmers in peri-urban villages such as Garchuk and Nagaom around Guwahati reported that veterinarians were reluctant to make in-person diagnoses due the time taken to travel to and from remote farms. This suggests that access—to veterinarians in general and, when veterinarians are available to their access to laboratories—is a serious issue that needs to be further addressed. A shortage of veterinarians results in over prescription and/or irrational AMU by untrained caregivers or informal prescribers, known as “private doctors” among Indian dairy farmers (19). In India, like many other LMICs, veterinarians are not the only prescribers of ABX for animal use. Para-veterinarians and other untrained community members might be more accessible, charging less for consultations compared to veterinarians. Over-the-counter access of ABX, without prescriptions, and direct marketing of drugs to farmers are also common practices in India (15).

In Assam, the most frequently observed diseases in cattle, goats, and poultry are potentially associated with the lack of hygiene, biosecurity, and non-adherence to the vaccination schedule for endemic diseases. A high proportion of veterinarians reported AMU for cattle with respiratory health problems, mostly caused by viral infections, followed by mastitis. This was also reported in a previous study that identified AMU mostly for mastitis cases (15). This could be prevented by improving environment and animal hygiene, prober nutrition and dry cow therapy (33). Veterinarians are prescribing ABX listed as highest priority, critically important antimicrobials (HPCIA) by the World Health Organization (WHO). This confirms the results of previous studies, in India and other LMICs, in which veterinarians used Quinolones and 3rd generation Cephalosporins for animal treatment (9, 10). In India, 76.8 and 47.8% of veterinarians were found to prescribe Quinolones and 3rd generation Cephalosporins for dairy farms (9).

Among many factors influencing veterinarians' decisions for AMU, the economic status of livestock owners was a significant determinant, with veterinarians assessing the ability of farmers to pay before prescribing ABX. In Bangladesh, it was found that the farmer's economic condition highly influenced the selection of ABX for treatment of livestock and poultry (31). Such subjective assessments are common in veterinary practice where prescription has been shown to be determined by perceived ease of administration and compliance, willingness of pet owners to give ABX, as well as animal characteristics (24). Cost of ABX was typically a factor in areas of varying socioeconomic status (24). We also found that the manufacturer's instructions were ranked as “very important” in calculating the dose of ABX by only 6% of respondent veterinarians. Other studies have also identified non-adherence to manufacturers' instructions as a reason for indiscriminate AMU in the agriculture sector (34). These findings contradict the observations in Bangladesh where drug instructions had a high-to-moderate influence on AMU (31).

Our results indicated that all respondents agreed that AMR is a major human and animal health problem caused by over use and/or improper AMU. However, in practice veterinarians are still prescribing ABX without prober laboratory diagnosis or AST. As discussed above this is due to the lack of on-site diagnostic facilities. These findings are similar to those from other LMICs such as Bangladesh (31) but contradict observations in developed countries where the majority of veterinarians followed AST before AMU (30, 35). However, availability and affordability of diagnostic tools and other facilities are different in developed and LMICs. Veterinarians suggested a wide range of preventive measures like proper animal nutrition, hygiene, and vaccination for the most frequently occurring animal diseases in the study area. This would result in reducing AMU and AMR in livestock production. They also identified key stakeholders responsible for implementing these prophylactic measures. However, they were not willing to participate in future work aiming to collect qualitative and quantitative data for AMU in animals. This is due to the lack of strict guidelines and regulations of AMU and having no time to keep records. As indicated in the results, in addition to the treatment of diseased animals, veterinarians are burdened with many other tasks as conducting extension services on animal production and health, implementing various government schemes, and running surveillance. Veterinarians' negative attitude toward keeping records for AMU may be due to the difficulties of being able to adhere to recommendations and guidelines if they did; they are not unaware of the dangers of inappropriate AMU but face very real barriers to improving practice. Further studies are required to understand the reasons behind veterinarians' negative attitudes toward keeping medical records and how better AMU can be supported. Unfortunately, it was impossible to do so due to COVID-19 restrictions but future research should use ethnographic assessments and qualitative research methods such as focus group discussions and in-depth interviews with veterinarians and farmers to properly address the problem and co-design solutions. Mixed methods provide deep understanding of the pattern and reasons of AMU that would not be identified using quantitative surveys alone (36, 37).

## Conclusion

This study sought to provide insights on prescribing behavior and opinions of veterinarians regarding AMU and AMR. Although veterinarians are aware of AMR risks, the lack of diagnostic facilities in most veterinary clinics means that most prescribe ABX based on clinical symptoms. They are unaware of the existing national and state level regulations, guidelines, and strategies for AMR. Furthermore, there is a need for improved communication between policy makers and field veterinarians to bridge a gap between prescribing guidelines and clinical usage. Future initiatives should explore ways of better connecting all stakeholders with policy makers to drive the uptake and evaluation of key regulatory messages associated with key policies. To moderate AMU in livestock production in India and other LMICs with similar production systems, strategies should focus on: (1) enhancing communication and regular dissemination of updated AMU guidelines between relevant stakeholders; (2) CPD for veterinarians and other key stakeholders; and (3) provision of affordable and accessible veterinary services. In designing interventions to meet these objectives, attention need to be paid to the evaluation of their effectiveness. There is currently a conspicuous gap in the evidence on what works in different animal health systems including the role of veterinarians in the diagnostic, prescribing and treatment process. This is a significant challenge in under regulated dispensary settings in LMICs.

## Data Availability Statement

The raw data supporting the conclusions of this article will be made available by the authors, without undue reservation.

## Ethics Statement

This study was approved by the Human Ethical Review Committee (HERC), Royal (Dick), School of Veterinary Studies, The University of Edinburgh (Ref No. HERC_673_21). Local ethical approval was not acquired as there was no Ethical Committee for social science research in our partner's institute. However, a permission from the head of the Animal Husbandry and Veterinary Department (AHVD) in Assam to interview veterinarians. As interviews were conducted via telephone calls, oral consents were obtained after assurance of confidentiality and anonymity of respondents (University of Edinburgh).

## Author Contributions

ME contributed to the concept, survey design, data analysis, and writing up. JC, GG, and AT contributed to the research design and editing of article. BS and HS participated in preparing questionnaires and editing the article. BD piloted the questionnaires and collected the necessary data from the respondents. MH and BS provided the necessary logistics and overall supervision for executing the work. DM contributed to the concept and proof reading. All authors contributed to manuscript revision, read, and approved the submitted version.

## Funding

This work was funded by the UK Newton Fund awards co-funded by the Economic and Social Research Council grant numbers ES/S000186/1 and the Department of Biotechnology (DBT), Government of India (BT/IN/Indo-UK/AMR/06/BRS/2018-19). For the purpose of open access, the author has applied a Creative Commons Attribution (CC BY) licence to any Author Accepted Manuscript version arising from this submission.

## Conflict of Interest

The authors declare that the research was conducted in the absence of any commercial or financial relationships that could be construed as a potential conflict of interest. The reviewer SK declared a shared affiliation, though no other collaboration, with one of the author MH to the handling Editor.

## Publisher's Note

All claims expressed in this article are solely those of the authors and do not necessarily represent those of their affiliated organizations, or those of the publisher, the editors and the reviewers. Any product that may be evaluated in this article, or claim that may be made by its manufacturer, is not guaranteed or endorsed by the publisher.

## Footnotes

^1^ https://cdn.who.int/media/docs/default-source/antimicrobial-resistance/national-action-plan-on-amr-(india).pdf?sfvrsn=9f396e90_1&download=true

^2^ https://vetsustain.org/

^3^“*Establishment and Strengthening of Veterinary Hospital and Dispensaries”. Available online at: https://animalhusbandry.assam.gov.in/projects/livestock-health-and-disease-control-lhdc-programme*

^4^https://animalhusbandry.assam.gov.in/portlets/strategy-papers-of-ah-veterinary-department (Strategy Papers on Manpower & Human Resource Rationalization).
