# Supplementary material for: Factors Influencing Antibiotic Prescribing Behavior and Understanding of Antimicrobial Resistance Among Veterinarians in Assam, India
Source: Front Vet Sci. 2022 Apr 26;9:864813. doi: 10.3389/fvets.2022.864813 (PMC9087579; doi:10.3389/fvets.2022.864813)
Supplement: Supplementary file 1 [file Data_Sheet_1.docx]

**Supplementary Material**

*Supplementary Table 1: The most prevalent diseases and health conditions most frequently seen by veterinarians in cattle, buffalo, sheep, goat and pigs in Assam, India.*

| Most prevalent diseases | Number of Veterinarians | | | | |
| --- | --- | --- | --- | --- | --- |
|  | Cattle | Buffalos | Sheep | Goat | Pigs |
| Three day sickness | 2 | - | - | - | - |
| Anoestrus | 4 | 1 | - | - | - |
| Anorexia | - | 1 | 1 | - | - |
| Bacterial dermatitis | - | 1 | - | - | - |
| Babesia | 1 | - | - | - | - |
| Black quarter (BQ) | 5 | - | - | - | - |
| Bloat/colic | 3 | 1 | - | 2 | - |
| Brucellosis | 3 | - | - | - | - |
| Carbohydrate engorgement/ Ruminant impaction | 1 | - | - | 2 | - |
| Conjunctivitis | 1 | - | - | 7 | - |
| Cowpox | 1 | - | - |  | - |
| Debility | 2 | 1 | - | 2 | 1 |
| Deficiency disease | 2 | 1 | - | - | 1 |
| Downer cow syndrome | 1 | - | - | - | - |
| Dysentery/diarrhoea | 1 | - | - | - | 1 |
| Dystocia | 1 | - | 1 | 3 | - |
| **Foot & Mouth disease (FMD)** | **22** | **8** | - | - | **8** |
| Foot rot | 1 | - | - | 1 | - |
| Gynaecological problem | 5 | - | - | 1 | - |
| Haemorrhagic septicaemia (HS) | 4 | 1 | - | - | - |
| **Ketosis** | 1 | **6** | - | - | - |
| Laminitis | 1 | 3 | - | - | - |
| **Lumpy skin disease (LSD)** | **20** | 1 | - | - | - |
| **Mastitis** | **25** | 1 | - | 1 | 1 |
| Metabolic disorders | 2 | 1 | 1 | - | - |
| Milk fever/hypocalcaemia | 12 | - | - | - | 1 |
| Non-specific diarrhoea | 1 | - | - | - | - |
| **Parasitic diseases (PD)** | **37** | 3 | 1 | **21** | **5** |
| Poisoning | 3 | - | - | 2 | - |
| Post flood diarrhoea | 1 | 1 | - | - | - |
| Prolapse | 1 | - | - | - | - |
| Pyometra/pyometritis/Metritis | 2 | - | - | - | - |
| Repeat breeding | 5 | - | - | - | - |
| Reproductive problems | 1 | - | - | - | - |
| Respiratory distress/diseases | 2 | - | - | 5 | 1 |
| Retention of Placenta (ROP) | 4 | - | - | 5 | - |
| Skin disease | 1 | - | 1 | 1 | 1 |
| Theileria | 1 | 1 | - | - | - |
| Tuberculosis | - | 1 | - | - | - |
| Papilloma | - | 1 | - | 2 | - |
| **Listeriosis/Gid** | - | - | 1 | **12** | - |
| **Peste des petits ruminants (PPR)** | - | - | 1 | **20** | - |
| Enterotoxaemia([Angulo et al.](#_ENREF_2)) | - | - | 1 | 5 | - |
| **Orf** | - | - | 1 | **12** | - |
| Mange infestation | - | - | - | 1 | 1 |
| Tetanus | - | - | - | 2 | 1 |
| Abscess | - | - | - | 2 | 1 |
| Goat pox | - | - | - | 5 | - |
| Wart | - | - | - | 1 | - |
| Cyst | - | - | - | 1 | 1 |
| **Swine fever (African Swine Fever/Classical Swine Fever)** | **-** | **-** | **-** | **-** | **37** |
| Swine erysipelas | - | - | - | - | 3 |
| Piglet anaemia | - | - | - | - | 3 |
| Piglet fever | - | - | - | - | 2 |
| Hernia | - | - | - | - | 1 |

*Supplementary Table 2: The most prevalent diseases and health conditions most frequently seen by veterinarians in poultry in Assam, India.*

| Diseases/Health conditions | Number of veterinarians |
| --- | --- |
| **Newcastle disease** | **32** |
| ***E. Coil*/Colibacillosis** | **10** |
| Salmonellosis | 6 |
| Infectious bursal disease (IBD) | 5 |
| Fowl pox | 5 |
| Thiamine deficiency/Vitamin deficiency | 3 |
| Chronic respiratory disease (CRD) | 2 |
| Duck plague (duck viral enteritis) | 2 |
| Duck cholera | 1 |
| Infectious coryza | 1 |
| Debility/Dietary deficiency | 2 |
| Enteritis | 1 |
| Bacillary white diarrhoea | 1 |
| Metabolic disorder | 1 |
| Coccidiosis | 1 |
| Marek's disease | 1 |

*Supplementary Table 3: Potential preventive measures suggested by veterinarians for the most prevalent diseases/health problems in livestock in Assam, India*

| Preventive measures | Diseases/Health problems | Number of Veterinarians | | | | | |
| --- | --- | --- | --- | --- | --- | --- | --- |
|  |  | Cattle | Buffalos | Sheep | Goat | Pigs | Poultry |
| Balanced diet | Deficiency disease | 2 | - | - | - | - | 2 |
|  | Metabolic disease | 1 | 1 | 1 | - | - | - |
|  | Anoestrus | - | 1 | - | 1 | - | - |
|  | Bloat | - | - | - | 1 | - | - |
| Deworming | **Parasitic disease(PD)** | **34** | **8** | **2** | **20** | **5** | - |
|  | Diarrhoea | - | 1 | - | - | - | - |
|  | Mange infestation | - | - | - | 1 | - | - |
| Hygiene | Mastitis | 2 | - | - | - | - | - |
|  | Listeriosis | - | - | - | 1 | - | - |
|  | Piglet fever | - | - | - | - | 1 | - |
| Proper management | **Mastitis** | **14** | 1 | - | 3 | 1 | - |
|  | Repeat breeding | 1 | - | - | - | - | - |
|  | Metabolic disease | 1 | - | - | - | - | - |
|  | Gynaecological problem | 2 | - | - | - | - | - |
|  | Tuberculosis | - | 1 | - | - | - | - |
|  | Respiratory tract infection | - | - | - | 1 | - | - |
|  | Piglet anaemia | - | - | - | - | 3 | - |
| Seromonitoring and chemotherapy | Lumpy skin disease(LSD) | 1 | - | - | - | - | - |
| Regular vaccination | **FMD** | **24** | 4 | - | - | 1 | - |
|  | Milk fever | 1 | - | - | - | - | - |
|  | Black quarter(BQ) | 5 | - | - | - | - | - |
|  | Haemorhagic septicaemia(HS) | 3 | 3 | - | - | - | - |
|  | Brucellosis | 1 | - | - | - | - | - |
|  | Cow pox | 1 | - | - | - | - | - |
|  | **Peste des petits Ruminants(PPR)** | - | - | 1 | **20** | - | - |
|  | Orf | - | - | 1 | 6 | - | - |
|  | Enterotoxaemia([Angulo et al.](#_ENREF_2)) | - | - | 1 | 2 | - | - |
|  | Goat pox | - | - | - | 13 | - | - |
|  | **Classical Swine fever** | | | | | **24** | - |
|  | African swine fever | | | | | 6 | - |
|  | Swine erysipelas | | | | | 1 | - |
|  | **New castle disease** | | | | | | **32** |
|  | Chronic respiratory disease(CRD) | | | | | | 2 |
|  | Duck plague | | | | | | 5 |
|  | Fowl pox | | | | | | 4 |
|  | Marek’s disease | | | | | | 1 |

*Supplementary Table 4: Mean, mode and standard deviation (SD) for 5-likert scale questions (1= Not important; 2=Slightly important; 3=Fairly important; 4=Important; 5 =Very important) for factors affecting decisions to prescribe antibiotics and deciding the dose*

| Factors | Mean | Mode | SD |
| --- | --- | --- | --- |
| **Influencing the decision to prescribe antibiotics** | | | |
| Disease Condition/Severity of clinical symptoms | 4.46 | 5.00 | 0.613 |
| Results of laboratory diagnosis | 4.54 | 5.00 | 0.676 |
| Cost of the antibiotic | 3.80 | 4.00 | 0.728 |
| Animal’s owner request | 1.38 | 1.00 | 0.753 |
| Availability of ABX | 3.82 | 4.00 | 0.873 |
| Route of administration | 4.12 | 4.00 | 0.718 |
| Antibiotic Sensitivity Test ([Kahan et al.](#_ENREF_8)) | 3.42 | 3.00 | 1.162 |
| Ease of administration | 3.58 | 4.00 | 0.731 |
| Withdrawal period of ABX | 4.40 | 4.00 | 0.606 |
| Economic status | 4.48 | 5.00 | 0.614 |
| **Influencing the dose of antibiotic** | | | |
| Manufacturer instructions | 3.74 | 4.00 | 0.723 |
| Animal’s age | 4.34 | 4.00 | 0.626 |
| Animal’s weight | 4.96 | 5.00 | 0.198 |
| Severity of illness | 4.94 | 5.00 | 0.240 |
| My own experience | 4.12 | 4.00 | 0.627 |

*Supplementary Table 5: Mean, mode and standard deviation (SD) for 3-likert scale questions (1= disagree, 2= Neutral, 3= Agree and 99=Don’t know) for veterinarians’ perception toward antimicrobial use (AMU) and antimicrobial resistance (AMR)*

| Statement | Mean | Mode | SD |
| --- | --- | --- | --- |
| ABR/AMR is a major human health problem in India | 3.00 | 3.00 | 0.000 |
| ABR/AMR is a major animal health problem in India | 3.00 | 3.00 | 0.000 |
| ABX usage in animals can affect the health of consumer | 2.96 | 3.00 | 0.198 |
| ABX usage in animals should be reduced | 2.76 | 3.00 | 0.431 |
| ABX usage in humans should be reduced | 2.74 | 3.00 | 0.443 |
| Overuse of ABX can lead to antibiotic resistance | 3.00 | 3.00 | 0.000 |
| Many ABX are not effective at treating disease in animals | 2.48 | 3.00 | 0.762 |
| Improper usage of ABX results in high costs for farmers | 3.00 | 3.00 | 0.000 |
| Using ABX in animals may affect their effectiveness in humans | 2.96 | 3.00 | 0.198 |

*Supplementary Table 6: Mean, mode and standard deviation (SD) for 5-likert scale questions (1= Not important; 2=Slightly important; 3=Fairly important; 4=Important; 5 =Very important) for potential measures and key stakeholders to reduce excess use of antibiotics*

| Statement | Mean | Mode | SD |
| --- | --- | --- | --- |
| **Potential measures to control the excess use of Antibiotics/Antimicrobials** | | | |
| Improving animal housing | 4.86 | 5.00 | 0.405 |
| Eradicating diseases | 4.92 | 5.00 | 0.274 |
| Educating farmers | 4.90 | 5.00 | 0.303 |
| Restricting unnecessary treatment | 4.88 | 5.00 | 0.328 |
| Providing continued education to vets/para-vets | 4.90 | 5.00 | 0.303 |
| Introducing antibiotic use tax | 3.14 | 3.00 | 0.881 |
| Use of alternative therapy | 4.28 | 4.00 | 0.573 |
| Prohibiting usage of ABX as growth promoters | 4.42 | 5.00 | 0.785 |
| **The main/key stakeholders to control the excess use of Antibiotics/Antimicrobials** | | | |
| Central government | 4.88 | 5.00 | 0.385 |
| State government | 4.84 | 5.00 | 0.370 |
| Farmers | 4.24 | 5.00 | 0.847 |
| Veterinarians | 4.88 | 5.00 | 0.328 |
| Para-veterinarians | 4.44 | 5.00 | 0.577 |
| Pharmaceutical companies | 3.98 | 4.00 | 0.589 |
| Pharmacies | 3.92 | 4.00 | 0.665 |
| Veterinary products shops | 3.90 | 4.00 | 0.814 |
| Dairy or meat retail shops | 3.84 | 4.00 | 1.131 |
| Livestock product exporters | 3.90 | 4.00 | 1.093 |
| Consumers | 4.30 | 4.00 | 0.839 |
